# Back Complaints in the Elders (BACE); design of cohort studies in primary care: an international consortium

**DOI:** 10.1186/1471-2474-12-193

**Published:** 2011-08-19

**Authors:** Jantine Scheele, Pim AJ Luijsterburg, Manuela L Ferreira, Chris G Maher, Leani Pereira, Wilco C Peul, Maurits W van Tulder, Arthur M Bohnen, Marjolein Y Berger, Sita MA Bierma-Zeinstra, Bart W Koes

**Affiliations:** 1 Department of General Practice, Erasmus MC, University Medical Center Rotterdam, the Netherlands; 2 The George Institute for Global Health, University of Sydney, Sydney, Australia; 3 Physical Therapy Department, Federal University of Minas Gerais, Belo Horizonte, Brazil; 4 Department of Neurosurgery, Leiden University Medical Center, Leiden, the Netherlands; 5 Department of Health Sciences & EMGO Institute for Health and Care Research, Faculty of Earth & Life Sciences, VU University Amsterdam, the Netherlands; 6 Department of General Practice, University Medical Center Groningen, Groningen, the Netherlands; 7 Department of Orthopaedics, Erasmus MC, University Medical Center Rotterdam, the Netherlands

## Abstract

**Background:**

Although back complaints are common among older people, limited information is available in the literature about the clinical course of back pain in older people and the identification of older persons at risk for the transition from acute back complaints to chronic back pain.

The aim of this study is to assess the course of back complaints and identify prognostic factors for the transition from acute back complaints to chronic back complaints in older people who visit a primary health care physician.

**Methods/design:**

The design is a prospective cohort study with one-year follow-up. There will be no interference with usual care. Patients older than 55 years who consult a primary health care physician with a new episode of back complaints will be included in this study.

Data will be collected using a questionnaire, physical examination and X-ray at baseline, and follow-up questionnaires after 6 weeks and 3, 6, 9 and 12 months.

The study 'Back Complaints in the Elders' (BACE) will take place in different countries: starting in the Netherlands, Brazil and Australia. The research groups collaborate in the BACE consortium. The design and basic objectives of the study will be the same across the studies.

**Discussion:**

This consortium is a collaboration between different research groups, aiming to provide insight into the course of back complaints in older people and to identify prognostic factors for the transition from acute back complaints to chronic back complaints in older persons. The BACE consortium allows to investigate differences between older people with back complaints and the health care systems in the different countries and to increase the statistical power by enabling meta-analyses using the individual patient data. Additional research groups worldwide are invited to join the BACE consortium.

## Background

Back pain is the most common musculoskeletal complaint seen in primary care. A systematic review of prevalence studies on low back pain found a point prevalence ranging from 12% to 33% [1]. According to van der Windt et al. 22.4% of the people with back complaints consults their general practitioner (GP) [2]. Studies including older people also show that back pain is a major problem in this population [3-5]. The most important features of back complaints are pain and disability. Older people with back complaints report difficulty with activities of daily living such as housework, shopping, walking and lifting objects [5,6]. Because of the high prevalence and consequences in terms of disability, health care costs associated with back pain are considerable. The total treatment costs of patients with back complaints in Australia exceed US$ 1 billion per year [7]. In the Netherlands, these costs range from €3.5 to 4.3 billion per year [8]. Between 1990 and 2020, it is estimated that the number of people aged 65 years and older will increase by 71% in most developed countries, implying that health care costs of older patients with back pain will increase substantially [9].

Although there are reports on the course of acute or subacute back complaints, few studies distinguished between younger adults and older persons [10-12]. Even when different age categories are compared, older people are under-represented and some studies explicitly exclude patients aged ≥ 60 or 65 years [11,12]. Therefore, little is known about the course of back complaints in older people, even though back complaints are a major health issue in this age group. A similar problem concerns with the identification of prognostic factors for the transition from acute back complaints to chronic back complaints in older people. Several studies have reported on prognostic factors, but the results are often contradictory [13] and none assessed these factors specifically in older patients.

It is important to assess the course of back complaints in older adults, because older age is frequently reported as a prognostic factor for the transition from acute back complaints to chronic back complaints [13]. This may indicate that older persons are more likely to have chronic back complaints. The prevalence of osteoarthritis, disc degeneration, osteoporosis and spinal stenosis are known to increase with increasing age [14,15], which can influence the course of back complaints. Older people also have more co-morbidity, which may influence the transition to chronic (back) pain. Prognostic research can help clinicians to identify patients at risk for chronic back complaints. Information on the course and prognosis is not only valuable for clinicians, but also informative for the patient. Mallen et al. reported that 82% of older people, visiting their GP with musculoskeletal pain found it important to be informed about the prognosis of their complaint by their GP [16].

If patients seek medical care for their back complaints, this usually takes place in a primary care setting. The GP evaluates the patient and decides whether further diagnostics and referral to secondary care or other health care providers are required. The diagnostics are, as recommended by several guidelines, mostly based on the presence of the 'red flags' as indicators of possible underlying pathology [17]. However, few studies have examined the diagnostic accuracy of these red flags. Henschke et al. [18] conducted a large cohort study to determine the presence of serious pathology when red flags were identified in people with an acute episode of back pain; they found that red flags usually present a high false-positive rate and only a few red flags (prolonged use of corticosteroids, age >70 years and significant trauma) were predictive for detecting fractures. No research on red flags and diagnostic interventions has been undertaken specifically in the older adult population. Before recommendations for use in clinical practice can be made, further evaluation of the red flags and diagnostic interventions is needed. In summary, there is a need to study the clinical course of back pain in the elders and to identify older people at risk for chronic back pain.

This cohort study will be set-up and conducted in different countries. Therefore, we established the BACE consortium to standardize methods regarding eligible patients and measurements. The consortium will allow us to compare the course and prognostic factors of back pain across different countries, and investigate the influence of healthcare systems on the treatment of back complaints. Meta-analysis using individual patient data will lead to more precise estimates of associations and opens the possibility to study outcomes in pre-defined subgroups of older patients with back pain.

The primary objectives of the cohort study are:

1) To determine the duration, severity and clinical course of back pain in older people who visit the GP with a new episode of back pain.

2) To identify possible prognostic factors for the transition from acute back complaints to chronic back complaints in older people.

Secondary objectives are:

1) To determine the level of functional disability, quality of life and productivity loss present in older people visiting their GP with back pain.

2) To establish the diagnostic value of the 'red flags' examined at baseline.

3) To determine the prevalence and prognostic value of the separate signs of vertebral degeneration and osteoporotic fractures in older people with back pain.

4) To determine the prevalence of underlying pathology (infection, tumor, fracture, radiculopathy, spondylarthritis) identified by the GP, in older people with back pain.

5) To determine the medical consumption of older people with back complaints, visiting their GP.

Additional objectives BACE consortium:

1) To identify differences regarding the course and prognostic factors of older people with back complaints visiting a GP in the different countries joining the BACE consortium.

2) To determine the impact of the different healthcare systems on the management of back complaints in older people.

3) To determine if prognostic factors found by national BACE studies can be validated in the BACE consortium.

4) To determine if meta-analysis using individual patient data of the different BACE studies leads to more precise estimates of associations.

5) To identify subgroups of older people with back complaints.

## Methods/design

### Design

**Figure 1:**
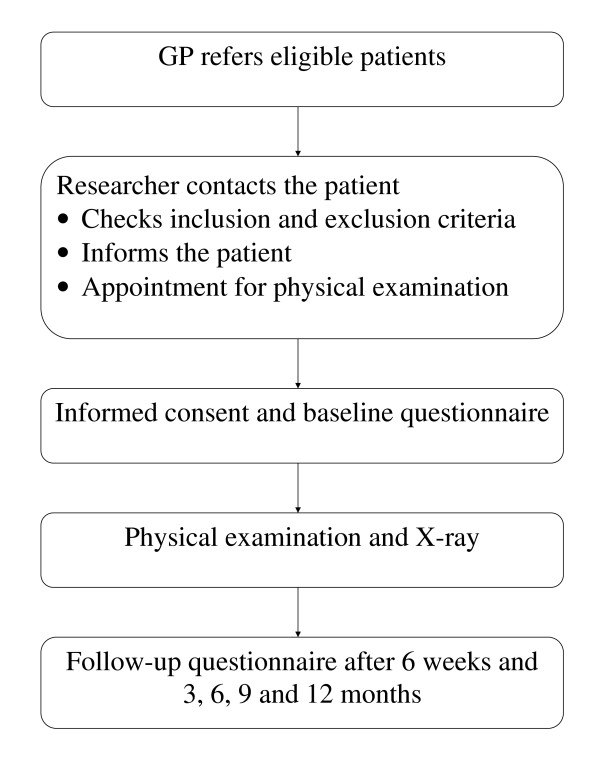
**Flow chart of the BACE study**.

This study will be a prospective cohort study with a follow-up period of one year. Data will be collected using questionnaires, physical examinations and X-ray examination. This study will be observational, meaning that there will be no interference with the care given by the GP or other healthcare providers with respect to advice, diagnostics or treatment. Before starting the study, the research protocol needs to be approved by the appropriate ethics committee of the different research groups joining the BACE consortium. This protocol has already received ethical approval from the Medical Ethics Committee of the Erasmus Medical Center, the Netherlands and the Ethic Committee in Research of Federal University of Minas Gerais, Brazil. Figure 1 presents a flowchart of this cohort study.

### Inclusion and exclusion criteria

Patients aged >55 years will be included in the BACE cohort when they consult a GP for a new episode of back complaints. All back complaints, defined as pain in the region from the top of the shoulder blades to the first sacral vertebra, will be included. An episode is considered 'new' if the patient has not visited a G P during the preceding 6 months for the same back complaint.

Patients who are unable to fill in the questionnaires as a result of language problems or a cognitive disorder will be excluded from the study, as are patients unable to undergo the physical examination (e.g. wheelchair-bound patients). An anonymous record will be kept of the number of patients who choose not to participate, as well as the number of the excluded patients and the reason for exclusion.

### Inclusion procedure

Participating GPs will be asked to refer all patients with a new episode of back pain, aged >55 years, to the BACE study. They will inform patients either during consultation or in writing within 2 weeks of their consultation. The GP will ask for the patient's permission to sent his/her contact information to the researchers. The researchers will contact the patients, answer any questions of the patient and make an appointment for the physical examination of those who verbally consent to participate. The informed consent procedure will be completed during the physical examination. The appointment for the X-ray examination will be made after the physical examination.

### Physical examination

A standardized protocol for the physical examinations has been developed by the researcher (JS) and two senior researchers with ≥ 10 years of experiences in both physiotherapy and research (SB-Z and PL). Trained research assistants will conduct the physical examination. Standardization of the examinations among the research assistants will be accomplished by a series of training sessions before commencing recruitment and will be repeated during the recruitment period. An instruction video and protocol will be available to ensure standardization between the different research groups joining the BACE consortium. The physical examination will be conducted as close to the GP consultation date as possible. During the physical examination some of the red flags will be measured. Other red flags will be assessed in the baseline questionnaire. Recorded red flags are based on the literature [19-23] and are summarized in Table 1.

**Table 1:** Red flag conditions indicating possible underlying spinal pathology or nerve root problems that will be recorded.

Red flag	Possible underlying pathology
Previous history of cancer	Cancer
Age at unset < 20 or > 55 years	Cancer
Unexplained weight loss	Cancer
Pain at rest	Cancer
Non-mechanical pain	Cancer, vertebral infection
Systematically unwell	Cancer, vertebral infection
Increased C-reactive protein level	Cancer, vertebral infection
Fever	Vertebral infection
Urine tract infection or skin infection	Vertebral infection
Recent bacterial infection e.g. urinary tract or skin infection	Vertebral infection
Age > 70 years	Fracture
Trauma as cause of the back complaint	Fracture
Sudden decrease in height	Fracture
History of osteoporosis	Fracture
Urinary retention	Cauda equina syndrome
Acute onset of urinary retention or incontinence	Cauda equina syndrome
Morning stiffness	Inflammatory disorder
Pain improves with physical activity	Inflammatory disorder
Pain in the leg worse than back pain	Lumbosacral radicular syndrome

The examination will consist of the following parts: 1) history taking e.g. pain location, severity of the pain, radiation of the pain and history of back pain, 2) inspection of the body e.g. palpation, neuropathic pain diagnostic questionnaire (DN4), ankle tendon reflex, knee tendon reflex and hypesthesia or hypalgesia of the foot and toes, 3) range of motion and additional diagnostic tests, e.g. test of Lasègue, finger-floor distance, muscular strength of the quadriceps muscle and the bone quality of the heel, measured with a quantitative ultrasound system (the Lunar Achilles InSight) [24,25]. Table 2 presents details of the physical examination. The patients will be blinded for the outcomes of the physical examination. If the information gathered during the physical information is important for the health of the patient, the GP will be informed (e.g. low bone quality or high C-reactive protein level). The physical examination will be performed only at baseline, to establish the characteristics of the complaints and to collect data on potential prognostic factors.

**Table 2:** Item list for physical examination.

History taking	Inspection	Range of motion and additional diagonistic tests
- Pain location	- Standing posture	- Standing on heels and toes
- Radiation of the pain	- Scars or other abnormalities	- Finger-floor distance and the presence of flexion pain
- Severity of pain (11-point numeric rating scale)	- Heberden's and Bouchard's nodules	- Latero-flexion: range and pain (yes/no)
- Leg pain > back pain	- Palpation of the paravertebral muscles	- Upper body rotation: range and pain
- Paraesthesia of the foot and toes	- Palpation spinous processes and sacroiliac joint	- Muscular strength of the m. quadriceps
- Non-mechanical pain	- Ankle tendon reflex	- Test of Lasègue [47,48]
- Neuropathic pain questions (DN4) [46]	- Knee tendon reflex	- Crossed test of Lasègue [47,48]
- History of back pain	- Hypesthesia or Hypalgesia of the foot and toes	- Exo- and endorotation of the hip: range and pain
- Pain and activity	- Neuropathic pain tests (DN4) [46]	- Bone quality of the heel
- Pain during coughing or sneezing		- Timed Up and Go test [49]
- Weight loss		- C-reactive protein level (blood sample)
- Comorbidity: e.g. urinal problems, obstipation, diagnosis of osteoporosis		

### X-rays

An X-ray will be made of the lumbar spine from both the anterior-posterior view and the lateral view. If patients have complaints of the thoracic spine, both X-rays will also be made of the thoracic spine. The X-rays and the radiologic report(s) will be requested at the hospital. The X-rays will be scored on the following features:

1) Disc degeneration will be evaluated using the grading system proposed by Lane et al. [26], based on the presence and severity of osteophytes and vertebral narrowing. In this grading system, grade 0 = none; grade 1 = mild; grade 2 = moderate; and grade 3 = severe.

2) Spondylolysthesis will be scored if the intervertebral sliding is > 2 mm [27].

3) Osteoporotic fractures will be evaluated using the system designed by Genant et al. Using this system, fractures are subdivided into 3 grades depending on the percentage of height reduction of the vertebrae: grade1 = mild, grade 2 = moderate and grade 3 = severe [28]. All fractures are confirmed by an expert radiologist.

4) Degenerative scoliosis will be defined as a lateral spinal curvature with a Cobb angle of 10° or more [29].

### Questionnaires

The baseline questionnaires will be filled in by patients before or just after the physical examination. The follow-up questionnaires will be sent (by e-mail or postal) at 6 weeks and at 3, 6, 9, and 12 months after the patient completed the baseline questionnaire. The questionnaires include outcome measures and prognostic factors, and are based on the recommendations presented in the Multinational Musculoskeletal Inception Cohort Study (MMICS) Statement [30].

Table 3 shows the measurements in the BACE study.

**Table 3:** Content of the patient questionnaires

	**Baseline**	**6 weeks**	**3 months**	**6 months**	**9 months**	**12 months**
**Demographics**						
- Age	X					
- Gender	X					
- Ethnicity	X					
- Educational level	X					
- Marital status	X					

**Outcome measures**						
- Global Perceived Effect (GPE) [31,32]	X	X	X	X	X	X
- Severity of pain (11-point numeric rating scale) [33]	X	X	X	X	X	X
- Recurrence of back pain		X	X	X	X	X
- Disability: Roland Disability Questionnaire (RDQ) [36]	X		X	X	X	X
- Health-related quality of life: Short Form-36 (SF-36) [38]	X		X	X	X	X
- PRodisq and DISease Questionnaire (PRODISQ) [39]	X		X	X	X	X
- Back medication: name, frequency and prescription/over-the-counter *	X		X	X	X	X
- Consultation to health care professionals*	X		X	X	X	X
- Health care satisfaction [50]*	X		X	X	X	X

**Prognostic factors**						
- Duration, onset of symptoms, frequency, radiation, numbness, weakness [51]	X	X	X	X	X	X
- McGill pain drawing [52]	X					
- Morning stiffness of the back (subscale of the WOMAC [53])	X	X	X	X	X	X
- Pain response to activity and position (PRAP) [54]	X					
- Physical activity: International Physical Activity Questionnaire (IPAQ) [55]	X		X	X	X	X
- Smoking (pack years)	X					
- Alcohol use: AUDIT-C Questionnaire [56]	X					
- Comorbidity: Self-administered Comorbidity Questionnaire (SCQ) [57]	X					
- Quality of sleep, subscale of the Pittsburgh Sleep Quality Index (PSQI) [58]	X					
- Kinesiophobia: Fear Avoidance Beliefs Questionnaire (FABQ) [59]	X					
- Pain Catastrophizing: Pain Catastrophizing Scale- Dutch Version (PCS-DV) [60]	X					
- Back Beliefs Questionnaire (BBQ) [61]	X		X	X	X	X
- Expectations of recovery: 5-point Likert scale; completely pain free/more pain than ever.	X		X	X	X	X
- Satisfaction with the current physical condition [50]	X		X	X	X	X
- Emotional well-being: CES-D [62]	X					
- Job Satisfaction: 7-point Likert scale; extremely unsatisfied/extremely satisfied	X					
- Co-workers support (subscale of Job Content Questionnaire (JCQ), [63])	X					
- Physical workload: Dutch Musculoskeletal Questionnaire (DMQ) [64]	X					

* These measures are also prognostic factors

### Outcome measures

The outcome measures included in the study will be global perceived effect, severity of back pain, recurrence of the back complaint, disability, quality of life, productivity loss during follow-up, medical consumption, and final diagnosis given by the GP.

Global perceived effect (GPE) will be measured on a 7-point scale, ranging from 'completely recovered' to 'worse than ever' [31,32].

Severity of back pain will be measured on an 11-point numerical rating scale (NRS) [33] in which 0 represents 'no pain' and 10 represents 'the worst pain ever'. We will measure the severity of back pain twice: for the moment of filling in the questionnaire and average back pain in the last week.

Different measurements will be used to gain insight into the recurrence of back complaints: the duration of the complaint (in days) and the duration of the pain-free period (in days). To define recurrence of back pain the definitions proposed by Stanton et al. and De Vet et al. are used [34,35]: a return of back pain lasting at least 24 h with a pain intensity of >2 on an 11-point NRS (>20 mm on a 100 mm VAS) following a period of at least 30 days pain free. The level of disability will be measured using the Roland Disability Questionnaire (RDQ), in which the patient's score can range from 0 (no disabilities) to 24 (severe disability) [36]. Quality of life will be measured with the Short-Form 36 (SF-36). The SF-36 measures 8 dimensions: physical function; role-physical; bodily pain; general health; vitality; social function; role-emotional; and mental health. Each dimension is scored from 0 to 100; a higher score representing better health [37,38].

All patients with a paid job will also complete the Productivity and DISease Questionnaire (PRODISQ) [39], which includes questions about their job, work absenteeism and loss of productivity.

To determine medical consumption, we will record back pain medication and the number of consultations with different healthcare professionals.

To determine the presence of serious pathologies, which can become apparent over time, GPs are asked to fill in a short questionnaire about the diagnosis of the back complaints at one-year follow-up.

### Prognostic factors

The following potential prognostic factors will be measured in the questionnaires: 1) demographic characteristics e.g. age and gender, 2) characteristics of the compliant e.g. duration of the complaint, the perceived cause, pain response to activity and position, 3) baseline functional disability (RDQ), 4) lifestyle e.g. smoking and alcohol use, 5) comorbidity (Self-administered Comorbidity Questionnaire), 6) psychological factors e.g. kinesiophobia, pain catastrophizing, back beliefs, expectations of recovery, emotional well-being, 7) work-related factors e.g. physical workload, job satisfaction and co-workers' support and 8) received treatment due to the back complaints e.g. medication and number of consultations. We will also measure characteristics of the national health system of the different countries joining the BACE consortium (e.g. insurance form, present guidelines, availability off direct access to medical facilities).

### Sample size

Based on the literature, 26-45% of the older adult population with acute low back pain will develop chronic persistent back complaints [11,12,40,41].Therefore, it is estimated that at least 30% of the older adults that visit the GP with a new episode of back complaints will have chronic persistent complaints.

To identify prognostic factors by means of multivariate regression analysis, 750 older adults with a new episode of back pain need to be included. This group consists of about 225 patients (0.3 * 750) that will have chronic back complaints. A minimum of 10 patients with chronic back complaints are needed to produce stable estimates for each prognostic factor. The estimated size of 225 subjects with chronic complaints, allows for multivariate regression analysis including 22 variables. These sample size calculations concern the individual national BACE studies. Combining the cohort data will obviously increase the statistical power of the analysis.

### Statistical analyses

Insight into the duration, severity and clinical course of back pain in the elders will be provided using descriptive statistics. Furthermore, descriptive statistics will provide insight into the level of functional disability, quality of life, productivity loss, medical consumption and prevalence of underlying pathology and X-ray findings. To evaluate the diagnostic value of the 'red flags', the sensitivity and specificity of the red flags will be calculated.

To identify prognostic factors for the transition from acute back complaints to chronic back complaints, we will first assess which factors of the baseline questionnaire and the physical examination are associated with chronic back complaints. A binary logistic regression analysis will be performed with these factors. Chronic back complaints are defined as back complaints lasting more than 3 months [42,43]. Global perceived Effect (GPE) will be used to determine whether the patient has recovered. This variable will be dichotomized because this allows estimating odds ratios (OR), which are easier to interpret in clinical practice. The scores 'somewhat improved', 'stayed the same', 'somewhat worsened', 'strongly worsened', 'worse than ever' will be defined as 'not recovered'. The scores 'completely recovered' and 'strongly improved' will be defined as 'recovered'. Factors with p <0.1 in the univariate logistic regression analysis will be included in the multivariate logistic regression analysis.

### Consortium

The BACE study will be conducted in different countries: starting in the Netherlands, Brazil and Australia. The aims of this collaboration are: to perform individual patient data meta-analyses, to validate prognostic models, to investigate the effects of cultural, economic and health care system differences on the clinical course of back pain, and to investigate cross-cultural differences in the treatment of back complaints in older people. The design will be the same across the studies, and a common set of outcome measures and possible prognostic factors will be used. The physical and X-ray examinations will be standardized. Same recruitment strategies will be implemented and the same inclusion and exclusion criteria will be used. All statistical analyses will be performed with the data of the different research groups separately and, if applicable, also together.

The BACE study in the Netherlands (BACE-D [Dutch]) started recruiting patients in 2009 and plans to end recruiting in September 2011. The Brazilian study (BACE-B) has been funded and is currently in preparation and starts recruitment of patients in September 2011. The Australian study (BACE-A) is applying for research funding.

The Consortium aims to assist other international research groups in the use of this proposed protocol to allow further cross-cultural comparisons and increase statistical power by enabling meta-analyses using individual patient data.

### Additional national objectives within the consortium

#### Falling in older people

A recent Australian cross-sectional study described that older people reporting pain and pain-related disability were more likely to have fallen in the past 12 months than people not reporting pain [44]. Therefore the BACE-A study will also include questions about the level of independence, number of falls, frailty and fear of falling. For that reason, the follow-up duration is set at two years. The same approach will be used in the BACE-B study.

The additional objectives are:

1) To establish the two-year incidence of falls, loss of independence, hospitalization, and institutionalization in back pain patients.

2) To identify prognostic factors for falls, loss of independence, hospitalization and institutionalization in back pain patients.

#### Long-term follow-up (5-years)

The BACE-D study will extend the follow-up period to five years. The additional long-term follow-up questionnaires will be sent at the 2, 3, 4 and 5-year follow-ups and will be the same as the 12-month questionnaire. After 5 years of follow-up anterior-posterior and lateral X-rays will be made of the lumbar spine. X-rays of the thoracic spine will only be made if the patient has complaints in the thoracic spine at follow-up; these will be scored in the same way as the baseline X-rays.

## Discussion

This cohort study will provide insight into the course of back complaints in older people visiting their GP and aims to identify prognostic factors for the transition from acute back complaints to chronic back complaints in the elders. Research groups in the Netherlands, Australia and Brazil already collaborate in the BACE consortium. This collaboration will allows to investigate cross-cultural differences between older people with back complaints and to increase the statistical power by enabling meta-analyses using the individual patient data. It will also allow us to investigate the influence of the national health care systems on the course and treatment of patients with back complaints. People’s health can be influenced by several factors such as guidelines, availability of health care, form of insurance and insurance costs [45].

We invite other research groups worldwide to join the BACE consortium, if interested.

## Competing interests

The authors declare that they have no competing interests.

## Authors' contributions

PAJL, SMAB-Z, WCP, MWvT, AMB, MYB and BWK developed the original concept of the study and developed the design of the national BACE-D cohort study. BWK and CGM conceived the idea of an international BACE Consortium. CGM and MLF developed the design of BACE-A and BACE-B. JS participated in the design of the BACE-D cohort study, is the coordinator of BACE-D and prepared the manuscript. All authors have read and approved the final version of the article.

## Pre-publication history

The pre-publication history for this paper can be accessed here:

http://www.biomedcentral.com/1471-2474/12/193/prepub
